# 551. Burn Patients with Skin Substitutes and Impact of Hyperbaric Oxygen Therapy

**DOI:** 10.1093/jbcr/irag033.248

**Published:** 2026-04-06

**Authors:** Hollie A Shreve, Nicole M Kopari, Christopher Tsutsui

**Affiliations:** Leon S. Peters Burn Center, Fresno, CA; Leon S. Peters Burn Center, Fresno, CA; Community Regional Medical Center, Fresno, CA

## Abstract

**Introduction:**

Successful burn wound closure often requires temporary dermal skin substitutes (DS). DS are frequently used to optimize wound beds before autografting in burn patients. Prolonged intervals between DS placement and grafting increase infection risk, length of hospital stay, number of operations and cost. Hyperbaric oxygen therapy (HBO) may accelerate DS integration. However, data on it’s impact in burn patients are limited. We present our case series of five burn patients treated with dermal substitutes and adjunctive HBO at our ABA verified burn center.

**Methods:**

This is a five-patient case review from January 2024 to September 2025 at our ABA verified burn center. Patients who underwent DS placement followed by a course of at least 10 HBO sessions. Inclusion criteria include 18 years old, mentally alert, able to provide informed consent, and willingness to attempt all planned sessions. A total of 15 patients were evaluated for HBO following DS placement with 5 patients meeting our inclusion criteria.

**Results:**

Among the 5 HBO patients, mean age was 51.4 years (range 35–70); two male and three female; mean TBSA 21.2%. Comorbidities included 2 patients with type 2 diabetes mellitus and hypertension. At an estimated $17 000/day, the 5.6-day reduction equates to ≈$92 200 saved in burn-unit costs per patient. With the average HBO cost of $1000 per session this still equates to a total cost savings of $456 000.

**Conclusions:**

In this single-center case review, HBO in burn patients with dermal substitutes was associated with a shorter average interval to autografting compared with patients who did not complete the full HBOT course. This small case review also illustrates potential cost implications and lower days to grafting and avoidance of additional procedures. These findings support HBOT as a promising adjunct to DS therapy.

**Applicability of Research to Practice:**

These findings support HBO as a promising adjunct to DS therapy and justify further prospective studies to clarify which burn patients may derive the greatest clinical and economic benefit.

**Funding for the study:**

N/A.

